# Supplementary material for: Hybrid Models and Biological Model Reduction with PyDSTool
Source: PLoS Comput Biol. 2012 Aug 9;8(8):e1002628. doi: 10.1371/journal.pcbi.1002628 (PMC3415397; doi:10.1371/journal.pcbi.1002628)
Supplement: Text S4 — Complete source code for the PyDSTool package (version 0.88.120504). Includes API documentation and help files linking to web pages. This file is identical to the current public release on Sourceforge.net. (ZIP) [file pcbi.1002628.s004.zip › PyDSTool/html/PyDSTool.Toolbox.optimizers.tests-module.html]

xml version="1.0" encoding="ascii"?


PyDSTool.Toolbox.optimizers.tests


| Home | Trees | Indices | Help | PyDSTool |
| --- | --- | --- | --- | --- |

Package PyDSTool ::
Package Toolbox ::
Package optimizers ::
Package tests

[hide private]

[frames] | no frames]

# Package tests

source code


|  |  |
| --- | --- |
| Submodules | [hide private] |

- **PyDSTool.Toolbox.optimizers.tests.test\_powell**: *Class defining the Powell function*
- **PyDSTool.Toolbox.optimizers.tests.test\_quadratic**: *Class defining a quadratic function*
- **PyDSTool.Toolbox.optimizers.tests.test\_rosenbrock**: *Class defining the Rosenbrock function*

  


| Home | Trees | Indices | Help | PyDSTool |
| --- | --- | --- | --- | --- |

|  |  |
| --- | --- |
| Generated by Epydoc 3.0.1 on Fri May 4 15:24:05 2012 | http://epydoc.sourceforge.net |
